# Membrane Fouling Behavior of Forward Osmosis for Fruit Juice Concentration

**DOI:** 10.3390/membranes11080611

**Published:** 2021-08-11

**Authors:** Zihe Li, Chongde Wu, Jun Huang, Rongqing Zhou, Yao Jin

**Affiliations:** 1 College of Biomass Science and Engineering, Sichuan University, Chengdu 610065, China; lhdyml@163.com (Z.L.); wuchongde@163.com (C.W.); Huangjun@scu.edu.cn (J.H.); zhourqing@scu.edu.cn (R.Z.); 2 Key Laboratory for Leather and Engineering of the Education Ministry, Sichuan University, Chengdu 610065, China; 3 National Engineering Research Center of Solid-State Manufacturing, Luzhou 646000, China

**Keywords:** forward osmosis, fruit juice concentration, xDLVO, membrane fouling

## Abstract

Forward osmosis (FO) technology has a broad application prospect in the field of liquid food concentration because of the complete retention of flavor components and bioactive substances. Membrane fouling is the main obstacle affecting the FO performance and concentration efficiency. This work systematically investigated the membrane fouling behavior of the FO process for fruit juice concentration elucidated by the models of resistance-in-series, xDLVO theory and FTIR analysis. The results show that the AL-FS mode was more suitable for concentrating orange juice. Increasing the cross-flow rate and pretreatment of feed solutions can effectively improve the water flux and reduce the fouling resistance. The ATR-FTIR analysis revealed that the fouling layer of orange juice was mainly composed of proteins and polysaccharides, and the pretreatment of microfiltration can greatly reduce the content of the major foulant. There was an attractive interaction between the FO membrane and orange juice foulants; by eliminating those foulants, the microfiltration pretreatment then weakened such an attractive interaction and effectively prevented the fouling layer from growing, leading to a lower process resistance and, finally, resulting in a great improvement of concentration efficiency.

## 1. Introduction

The large volume and perishability of liquid food cause the high expenditures of the packaging, storage, and transportation, which limits the consumption of liquid food. Therefore, concentration technology is crucial in liquid food processing. The application of the forward osmosis process in food concentration is a research hotspot in recent years [1,2]. Compared with other mainstream concentration technologies, such as thermal concentration, its biggest advantage is that the flavor components and bioactive substances in concentrated liquid food can be completely retained [3]. The recent review literature shows that forward osmosis has a broad application prospect in ensuring the original sensory and nutritional value and in the production of concentrated fruit juice with a high quality and high added value [1,4].

Forward osmosis is an osmotic pressure driven process, in which the solvent is transported from the feed solution (FS) to the draw solution (DS) through a semi-permeable membrane [5]. Many studies have concluded that compared with the reverse osmosis (RO) process, the FO process has a better water recovery, lower membrane fouling propensity and much better energy efficiency [6]. The advantages of the FO process are low-energy consumption and low concentration polarization on the membrane surface, i.e., lesser membrane fouling than RO [6,7,8]. The FO process has been successfully studied in various applications such as wastewater recovery [9,10], seawater desalination [11], power generation and food processing [5].

Although forward osmosis shows a lower tendency of fouling compared with other pressure-driven membrane processes, the inevitable membrane fouling is still the main obstacle affecting the performance and concentration efficiency of forward osmosis [12,13]. The membrane–foulant interaction leads to the adsorption of foulant on the surface of the FO membrane, and the foulant–foulant interaction leads to the accumulation of foulant on the surface of the FO membrane. Fouling formation depends on various parameters, such as operation parameters [14,15,16,17], membrane properties [18,19] and feed characteristics [20]. Therefore, the identification of membrane fouling behavior for each specific circumstance is necessary to offer better guidance for the process control of FO.

In the aspect of FO concentration of liquid food, pioneers have contributed great efforts to the degree of concentration, biological activity and the retention of flavor substances. The total soluble solids (TSS) of red radish extract [21] were increased by the FO process from 1.2 °Bx to 5.1 °Bx (4.25 folds) in a single concentration process and to 8.2 °Bx (6.83 folds) in a double concentration process. The double FO concentration increased the anthocyanin content in the concentrated juice from 0.258 to 0.899 mg/mL (3.48-fold). Moreover, the anthocyanin contents in beetroot and grape juices [22] were enriched to 2.91 g/L (57-fold) and 715.6 mg/L (6.8-fold), respectively. FO-concentrated anthocyanin in rose petal juice [23] showed the least change and could be stored for up to 2 weeks compared with liquid nitrogen treatment, ultrasonic treatment and freeze–thawing. A high antioxidant-concentrated Jaboticaba juice [24] with preserved activities and concentrated raspberry juice [25] with no significant changes in aroma and flavor is produced by the FO process. 

The advantages of FO in food concentration provide sufficient motivation for the study of membrane fouling behavior in this field. However, our current understanding of such a process is far from complete. To the best of our knowledge, there are few studies focusing on the fouling behavior of FO in food concentration. By now, the evolution of the water flux and membrane autopsy are nearly the only aspects being investigated that involved membrane fouling behavior. No comprehensive study can be found in the literature on liquid food concentration by FO integrally taking into account the FO performance, process resistance, foulant/membrane interaction and foulant identification.

Based on these considerations, this paper encompasses an integral investigation into the fouling behavior of FO concentration in food. The effects of different operating conditions and pretreatments on FO performance and membrane fouling are explored. The identification and interaction of membrane fouling are evaluated. To the best of our knowledge, this is the first time that the membrane fouling behavior of the FO process for fruit juice concentration was systematically investigated, providing a global understanding of membrane fouling in the process of FO for liquid food concentration; thus, offering guidance for process improvement.

## 2. Materials and Methods

### 2.1. Working Solutions and Membranes

The draw solution was 2 mol/L sodium chloride (analytical grade, Jinshan Chemicals, Shifang, China) solution. The orange juice used in the experiment came from NFC (Not from Concentrate) orange juice (Nongfu Spring, Hangzhou, China). Raw orange juice (ROJ) was the commercial juice that was filtered out of the added flesh. Centrifugal orange juice (CeOJ) was obtained by centrifuging the raw orange juice under a relative centrifugal force of 3000 g (LG-10M, Shuke, Chengdu, China) for 10 min. The microfiltration orange juice (MFOJ) was obtained by filtering the raw orange juice on 0.5 μm ceramic membrane (Filter & Membrane Technology Co. Ltd., Xiamen, China).

In this study, a flat-sheet cellulose triacetate–polyester (CTA-ES) FO membrane was used which was purchased from HTI (Hydration Technology Innovations, Albany, CA, USA). The membrane is a typical asymmetric CTA-ES membrane consisting of a thin active layer of cellulose triacetate and a thick support layer of polyester mesh, providing selective and mechanical support for the membrane, respectively. Its basic parameters were as follows [26,27]: interception rate (NaCl): 97%; membrane material: CTA; pH range: 3~8; operating temperature: 5~60 °C; water permeability coefficient: 0.98 L/(m^2^h·bar).

### 2.2. Experimental Protocol

**Figure 1:**
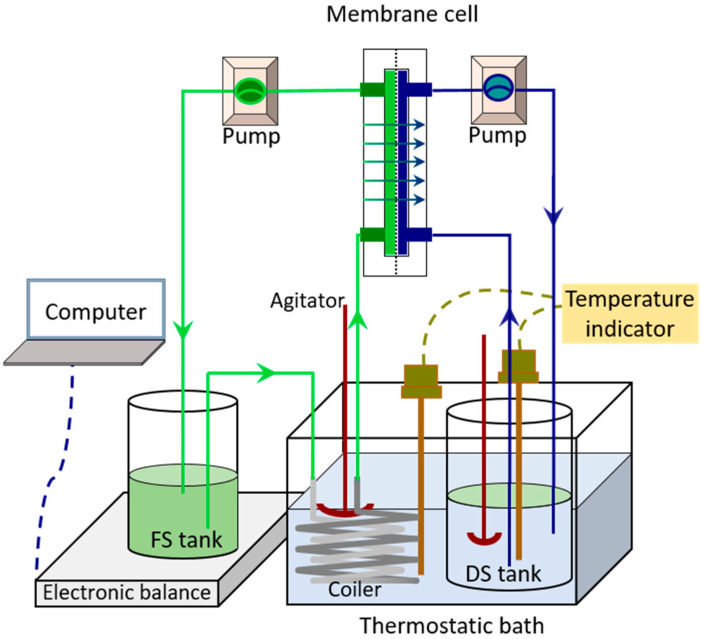
Schematic diagram of forward osmosis concentration experimental device.

Our previous work [17] developed a laboratory-scale FO unit utilizing a flat-sheet cellulose triacetate (CTA) membrane, as shown in Figure 1. A membrane cell with an effective membrane area of 0.003315 m^2^ was used. Two peristaltic pumps (BT600SV2-CE, Lead Fluid Technology Co., Ltd., Baoding, China. Pump head: YZ15; tubing size: 25#) were used to continuously inject feed and draw solution into the membrane cell. The temperature of both feed and draw solution was maintained constant before entering the membrane cell by a thermostatic bath (SDC-6, SCIENTZ, Co. Ltd., Ningbo, China). An electronic balance with an accuracy of 0.01 g (CP4102, Ohaus, Co. Ltd., NJ, USA) connected to a computer was used to record the mass variation in the feed tank over time (every 1 min).

The forward osmosis process was carried out at 20 °C. The draw solution and feed solution were 2 L sodium chloride solution and 800 g orange juice, respectively. When the effect of membrane orientation (AL-FS mode and AL-DS mode) was investigated, the forward osmosis equipment used ROJ as the FS and ran at the shear rate of 6.4 m·s^−1^ for 10 h. When the effect of shearing rate (6.4 m·s^−1^, 9.6 m·s^−1^ and 12.8 m·s^−1^) was investigated, the forward osmosis equipment ran in AL-FS mode for 10 h with ROJ as the FS. When exploring the effect of pretreatment, the forward osmosis equipment ran at the shear rate of 6.4 m·s^−1^ in AL-FS mode for 24 h with ROJ, CeOJ or MFOJ as the FS.

### 2.3. Analysis of FO Performance and Fouling

The FO performance was determined by water flux and concentration factor, and the calculation formula is as follows:

(1) $$J=\frac{\Delta m/\rho }{A_{m}\times \Delta t}$$

where *J* is the water flux (L·m^−2^·h^−1^ or LMH); Δ*m* (g) is the mass change of FS in Δ*t* (h); the density of water ρ is 1000 g/L; *A_m_* is the effective membrane area (m^2^).

(2) $$CF=\frac{m_{0}}{m_{t}}$$

where *CF* is the concentration factor; *m*_0_ and *m_t_* are the mass of the FS at the running time of 0 and t.

The resistance in a series model was applied to evaluate the fouling characteristics of the FO membrane. According to this model, the water flux, *J*, can be expressed as follows [28,29]:

(3) $$J=\frac{\Delta \pi }{\mu R_{t}}=\frac{\Delta \pi }{\mu \left(R_{m}+R_{c}+R_{p}\right)}$$

where Δ*π* is the transmembrane pressure (Pa); *μ* is the viscosity of the permeate (Pa·s); *R_t_* is the total resistance (m^−1^); *R_m_* is the resistance (m^−1^) due to membrane itself and dilutive concentration polarization of draw solute; *R_c_* is the fouling resistance (m^−1^) due to cake layer; *R_p_* is the fouling resistance (m^−1^) due to pore plugging. 

The experimental procedure to measure each of the resistance values was as follows: (i) *R_m_* was obtained through the average membrane flux of the clean membrane in the first hour using 2 mol/L NaCl as DS and UP water as FS. (ii) After the concentration at the corresponding time, the concentrated FS and diluted DS were replaced by UP water and fresh 2 mol/L NaCl solution. R_t_ was then measured from the average flux of the used FO membrane in first hour. (iii) Then, the membrane was removed and flushed with UP water to remove the cake layer. After this cleaning step, the water flux of the used membrane was measured again for one hour using 2 mol/L NaCl solution as DS to obtain the sum of *R_m_* + *R_p_*. The fouling resistance, *R_c_* was obtained by subtracting *R_m_* + *R_p_* from *R_t_*. Finally, the fouling resistance due to pore plugging *R_p_* was then obtained by subtracting *R_c_* and *R_m_* from *R_t_*. The instantaneous resistance was also calculated by Equation (3) and the change of osmotic pressure difference could be calculated according to dehydration volume.

The characteristic of the clean membrane and the fouling layer was analyzed through the Attenuated Total Reflection-Fourier Transform Infrared Spectroscopy (ATR-FTIR), and spectra results were obtained in the 4000~400 cm^−1^ region (Nicolet IS10, Thermo Scientific, Waltham, MA, USA).

### 2.4. Analysis of Foulants/Membrane Interaction

#### 2.4.1. Surface Tension Determination

The surface tension (*γ*) of solid samples was determined according to the theory of van Oss–Chaudhury–Good (vOCG) [30,31,32] based upon contact angle measurement of probe liquids. Such theory divided *γ* into nonpolar Lifshitz–van der Waals forces *γ^LW^* and polar Lewis acid–base component *γ^AB^*:

(4) $$\gamma =\gamma^{LW}+\gamma^{AB}$$

where *γ^AB^* can be expressed as the function of solid or liquid electron donor *γ*^−^ and electron acceptor *γ*^+^:

(5) $$\gamma^{AB}=2\sqrt{\gamma^{+}\gamma^{-}}$$

The clean or fouling membrane was dried for 24 h and the fruit juice was pressed into tablets after freeze-drying (LGJ-1, Yatai Kelong, Beijing, China), which were samples to be tested. The contact angle *θ* of samples measured by the optical contact angle tester (OCAH200, DataPhysics, Germany) had the following relationship with the surface tension parameters:

(6) $$\gamma_{l}\left(1+cos\theta \right)=2\left(\sqrt{\gamma_{s}^{LW}\gamma_{l}^{LW}}+\sqrt{\gamma_{s}^{+}\gamma_{l}^{-}}+\sqrt{\gamma_{s}^{-}\gamma_{l}^{+}}\right)$$

where the subscripts “*l*” and “*s*” refer to the liquid and solid phases, respectively. For the probe liquids (ultra-pure water, formamide and diiodomethane), all the tension component values were referred to in the literature [33], then the surface tension parameters of solid were deduced by solving the ternary equation, delivering the corresponding surface tension in combining with Equations (4) and (5).

#### 2.4.2. Interfacial Free Energy Determination

The membrane–foulant interactions could be described by extended Derjaguin–Landau–Verwey–Overbeek (XDLVO) theory [34], which describes the total interaction energy per unit area of particle–surface in terms of Lifshitz–van der Waals (*LW*), electrostatic (*EL*) and acid–base (*AB*) interaction energy [30], written as:

(7) $$\Delta G^{TOT}=\Delta G^{LW}+\Delta G^{AB}+\Delta G^{EL}\;$$

The Lifshitz–van der Waals, polar (acid–base) interfacial free energy and electrostatic double-layer interaction (Δ*G^LW^*, Δ*G^AB^* and Δ*G^EL^*) per unit area, could be calculated by the surface tension parameters of the membrane, foulant and water, which is showed in the following Equations (8)–(10):

(8) $$\Delta G^{LW}=2\left(\sqrt{\gamma_{l}^{LW}}-\sqrt{\gamma_{m}^{LW}}\right)\left(\sqrt{\gamma_{f}^{LW}}-\sqrt{\gamma_{l}^{LW}}\right)\;$$

(9) $$\Delta G^{AB}=2\sqrt{\gamma_{l}^{+}}\left(\sqrt{\gamma_{m}^{-}}+\sqrt{\gamma_{f}^{-}}-\sqrt{\gamma_{l}^{-}}\right)+2\sqrt{\gamma_{l}^{-}}\left(\sqrt{\gamma_{m}^{+}}+\sqrt{\gamma_{f}^{+}}-\sqrt{\gamma_{l}^{+}}\right)\;$$

(10) $$\Delta G^{EL}=\frac{\varepsilon_{0}\varepsilon_{r}\kappa }{2}\left(\zeta_{m}^{2}+\zeta_{f}^{2}\right)\left(1-\cot h\left(\kappa d_{0}\right)+\frac{2\zeta_{m}\zeta_{f}}{\left(\zeta_{m}^{2}+\zeta_{f}^{2}\right)}\mathrm{csch}\left(\kappa d_{0}\right)\right)\;$$

where the subscripts “*m*”, “*l*” and “*f*” corresponds to membrane, liquid and foulant, respectively. *ε*_0_ is the permittivity of free space, ε_r_ the dielectric constant, and *ζ_m_* and *ζ_f_* are the surface potentials of the membrane and foulant, respectively. *к* is the inverse Debye screening length [30]. When the interfacial free energy of the two surfaces was the same membrane or the same foulant, the parameters in the formula were replaced by the tension parameters of the same substance.

Zeta potential of foulant (*ζ_f_*) was measured with Malvern Mastersizer (ZEN3600 + MTP2, Malvern, UK).

Zeta potential of membrane (*ζ_m_*) at the membrane surface was performed using Electro Kinetic Analyzer (Anton Paar Surpass 3) determined from the measurement of the streaming potential using adjustable gap cell.

#### 2.4.3. Interfacial Interaction Energy

The interfacial interaction between two substances in solution consists of van der Waals interaction (*LW*), polar interaction (*AB*) and electrostatic interaction (*EL*) [30]:

(11) $$U^{TOT}=U^{LW}+U^{AB}+U^{EL}$$

*U^TOT^* (kT) is a function of distance which represents the total interfacial interaction energy between two substances in solution. In order to get close to the interface free energy between membrane and foulant, the two interfaces can be regarded as spherical particle interface and facial film interface, which can be calculated by Derjaguin approximate integral, and the expression of its component is as follows:

(12) $$U_{mlf}^{LW}\left(d\right)=2\pi d_{0}^{2}\Delta G^{LW}\left(\frac{a}{d}\right)$$

(13) $$U_{mlf}^{AB}=2\pi a\lambda \Delta G^{AB}\exp\left(\frac{d_{0}-d}{\lambda }\right)\;$$

(14) $$U_{mlf}^{EL}=\pi a\varepsilon_{r}\varepsilon_{0}\left(2\zeta_{m}\zeta_{f}\ln\left(\frac{1+\exp\left(-\kappa d\right)}{1-\exp\left(-\kappa d\right)}\right)+\left(\zeta_{m}^{2}+\zeta_{f}^{2}\right)\ln\left(1-\exp\left(-2\kappa d\right)\right)\right)$$

where *a* is the hydraulic radius (nm) of foulant particles; *d* is the distance (nm) between two interacting planes; the balance distance *d*_0_ is 0.158 nm; the characteristic attenuation length *λ* of polar force in aqueous solution is 0.6 nm.

By using the Derjaguin integral method, the interfacial interaction between the surfaces of foulants can be regarded as the interaction between spherical particles. The formulas for calculating the interaction energy of van der Waals force, polar force and electrostatic force are as follows:

(15) $$U_{flf}^{LW}\left(d\right)=2\pi d_{0}^{2}\Delta G^{LW}\left(\frac{a_{1}a_{2}}{d\left(a_{1}+a_{2}\right)}\right)$$

(16) $$U_{flf}^{AB}=\frac{2\pi a_{1}a_{2}}{a_{1}+a_{2}}\lambda \Delta G^{AB}\exp\left(\frac{d_{0}-d}{\lambda }\right)\;$$

(17) $$U_{flf}^{EL}=\pi \varepsilon_{r}\varepsilon_{0}\frac{a_{1}a_{2}}{a_{1}+a_{2}}\zeta_{f}^{2}\ln\left(1+\exp\left(-\kappa d\right)\right)\;$$

where *a*_1_ and *a*_2_ are the hydraulic radii of two spherical foulants. Hydraulic radius of foulant particles was measured with Malvern Mastersizer (ZEN3600 + MTP2, Malvern, UK).

## 3. Results and Discussion

### 3.1. FO Performance

In this section, orange juice was concentrated by the FO process, and its performance was investigated in different membrane orientations, cross-flow rates and pretreatments. The effect of the above-mentioned operating conditions on the water flux concentration factor and instantaneous resistance was revealed.

#### 3.1.1. Effect of Membrane Orientation

**Figure 2:**
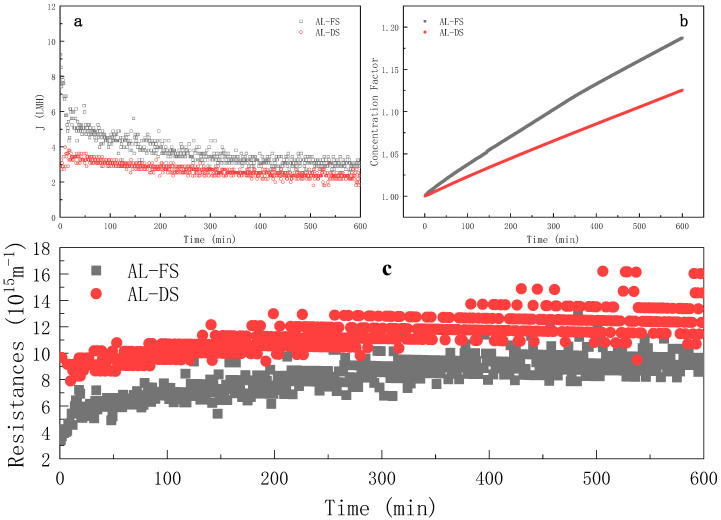
Evolution of water flux (**a**), concentration factor (**b**) and instantaneous resistance (**c**) over time during FO process at different membrane orientations (FS = ROJ; cross-flow rate = 6.4 m·s^−1^; t_R_ = 10 h).

Figure 2 illustrates the evolution of the water flux, concentration factor and resistance evolution of the FO process on different modes over time. As shown in Figure 2a, the water flux was generally higher on the AL-FS mode than on the AL-DS mode, which may a result from the blockage of fouling in the support layer on the AL-DS mode [35], such a phenomemon was already evidenced previously [17]. On the AL-FS mode, the initial flux of the ROJ was about 10 LMH and, then, the water flux dropped rapidly in the first 50 min. It can be seen from Figure 2c that the total resistance increased quickly at the same stage, probably due to the combinative effect of both the dilutive internal concentration polarization in the support layer side and the membrane fouling in the active layer side. This was mainly due to the rapid formation of membrane fouling. The analysis in Section 3.1.3 shows that the concentration polarization resistance was constant from 0 to 600 min, so the increase in total resistance was attributed to the increase in the fouling resistance. The water flux decreased slowly from 50 to 400 min as the above-mentioned phenomena tended to reach the steady state progressively.The water flux was approximately stable from 400 to 600 min which was only 31.9% of the initial flux, and the loss of water flux was serious.

As shown in Figure 2b, the concentration factor increased with the continuous progress of the forward osmosis process. At the 600th minute, the concentration factors were 1.187 (AL-FS) and 1.125 (AL-DS), respectively. As shown in Figure 2b, the concentration efficiency of the AL-FS mode was always higher than that of the AL-DS mode. When the established concentration factor was 1.125, the AL-FS mode could reach in 373 min, and AL-DS took 60% more time (that was, 226 min) to reach the same concentration factor.

#### 3.1.2. Effect of Cross-Flow Rate

**Figure 3:**
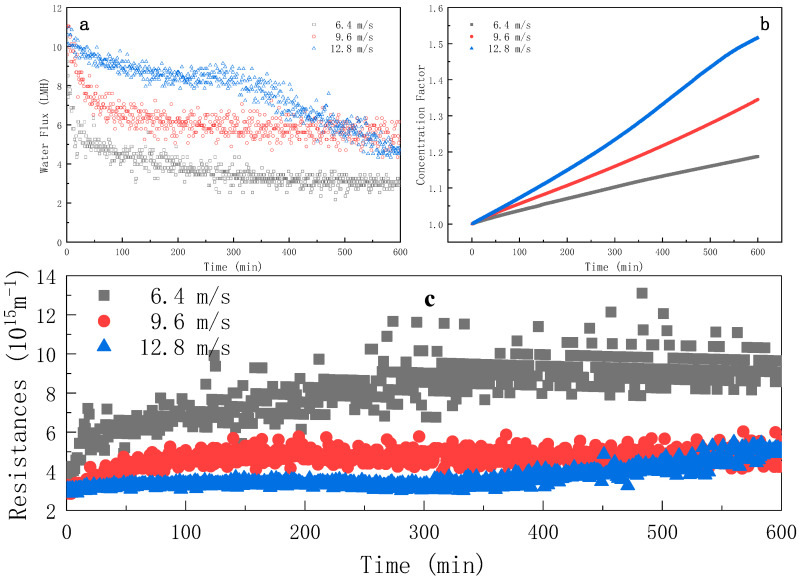
Evolution of water flux (**a**), concentration factor (**b**) and instantaneous resistance (**c**) over time during FO process at different cross-flow rates (FS = ROJ; AL-FS mode; t_R_ = 10 h).

The water flux evolution of Figure 3a indicates that the increasing cross-flow rate could obviously increase the water flux. Under the three cross-flow rates, the trend of water flux in the first 300 min was the same. The water fluxes decreased rapidly from 0 to 100 min; then, slowed down to reach a pseudo-steady state from 100 to 300 min. After 300 min, the water flux at the cross-flow rates of 6.4 m·s^−1^ and 9.6 m·s^−1^ tended to be stable, but that of 12.8 m·s^−1^ decreased abruptly. The resistance evolution in Figure 3c shows that the increase in cross-flow rate could effectively reduce the process resistance, which indicates that the additional shear force generated by the increased cross-flow effectively hindered foulants from accumulating on the membrane surface [14,15,16,17]. When the cross-flow rate was 12.8 m·s^−1^, the total resistance slightly increased from 0 to 300 min, followed by a sudden increase after 300 min; such a phenomenon will be further discussed in Section 3.1.3.

As shown in Figure 3b, the effect of the cross-flow rate on the concentration factor was important: the higher the cross-flow rate, the higher the concentation factor. The concentration factors at 600 min were 1.187, 1.345 and 1.516 for 6.4, 9.6 and 12.8 m·s^−1^, respectively. The time required to reach the established concentration factor 1.150 was 436, 282 and 200 min for 6.4, 9.6 and 12.8 m·s^−1^, respectively. Those results indicate that the concentration efficiency was improved with the increasing cross-flow rate resulting from the breakage of the fouling layer under enhanced shear stress at the membrane interface.

#### 3.1.3. Effect of Pretreatment

**Figure 4:**
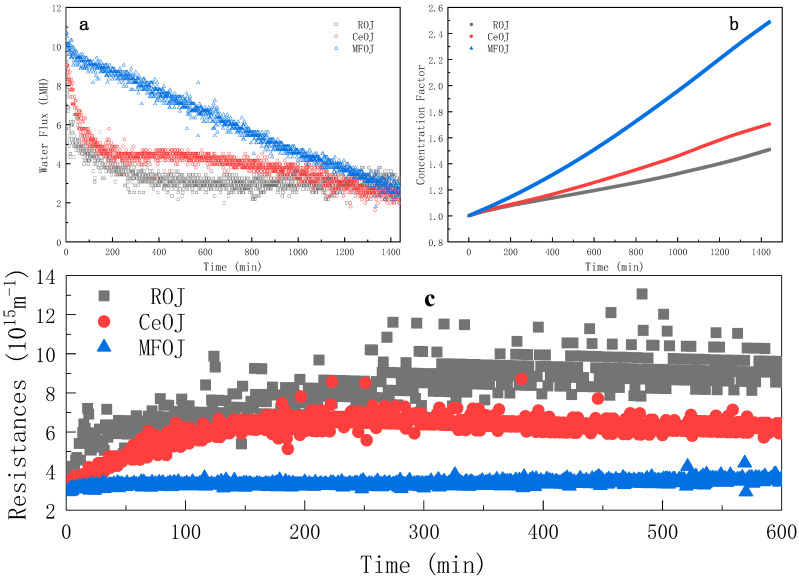
Evolution of water flux (**a**), concentration factor (**b**) and instantaneous resistance (**c**) over time during FO process after different pretreatments (cross-flow rate = 6.4 m·s^−1^; AL-FS mode; t_R_ = 24 h).

In this section, the feed solution was pretreated and concentrated in the AL-FS mode for 24 h in order to explore the main contributors of membrane fouling. Two different pretreatments were employed: centrifugation and microfiltration. The water flux evolution illustrated by Figure 4a suggests that both pretreatments can enhance the water flux during the FO process for orange juice concentration, which is consistent with the relevant work in the literature [20].

When the raw orange juice (ROJ) was used as the feed solution, the water flux was seriously lost in the first 300 min, which then became quasi-stable after 300 min. When the centrifugal orange juice (CeOJ) was used as the feed solution, the average water flux was slightly higher than that of the ROJ, while the evolution trend in the first 300 min was similar as that of the ROJ. The water flux was stable in 300–600 min and decreased slowly after 600 min.

It can be seen from Figure 4c that the water flux of the microfiltrated orange juice (MFOJ) decreased obviously in 0–300 min, but the total resistance was basically unchanged. The total resistance included the membrane resistance, fouling resistance and concentration polarization resistance. As indicated in Section 3.4, the MFOJ had removed the main pollutants, so the fouling resistance could be ignored, which implies that the concentration polarization resistance was basically constant from 0 to 600 min (only a slight increase at the beginning). It is then assumed that the decline of the water flux mainly resulted from the decrease in osmotic pressure difference, the driving force of FO in this circumstance.

Otherwise, an interesting “coincidence” was revealed by comparing the water flux evolution curve at the cross-flow rate of 12.8 m·s^−1^ (Figure 3) with that of the MFOJ (Figure 4). The evolution of both two curves was divided into two regimes: the gradual decrease until 300 min and an abrupt decrease at around 300 min; then, followed by a new decreasing regime. It can be speculated that there was no obvious membrane fouling in the first 300 min under these two experimental conditions thanks to the high cross-flow rate or microfiltration treatment. The main reason for the decrease in the water flux was the concentration polarization induced by the high water transfer. The concentration polarization increased the concentration of the feed solution on the surface of the active layer compared with the bulk. Along with the process running, the concentration of the feed on the membrane surface continuously increased. The increase in the concentration of the raw material on the surface of the active separation layer led to the increase in the concentration of pollutants. The sudden decrease in the water flux curve at 300 min may be attributed to the fact that the shear force was no longer strong enough to resist the establishment of a highly concentrated pollutant layer (either adhesive or not) to the membrane surface.

As shown in Figure 4b, the concentration efficiency was significantly improved by pretreatment, especially by microfiltration. For 24 h runs, the concentration factor reached 1.50, 1.71 and 2.49 for raw, centrifugal and microfiltration, respectively. Those results imply that the pretreatment by microfiltration was able to eliminate the major foulants of orange juice for the FO process; thus, to significantly enhance the process efficiency.

### 3.2. Resistance Distribution

In order to understand membrane fouling in the FO concentration of orange juice, the process resistances were measured after concentration.

**Figure 5:**
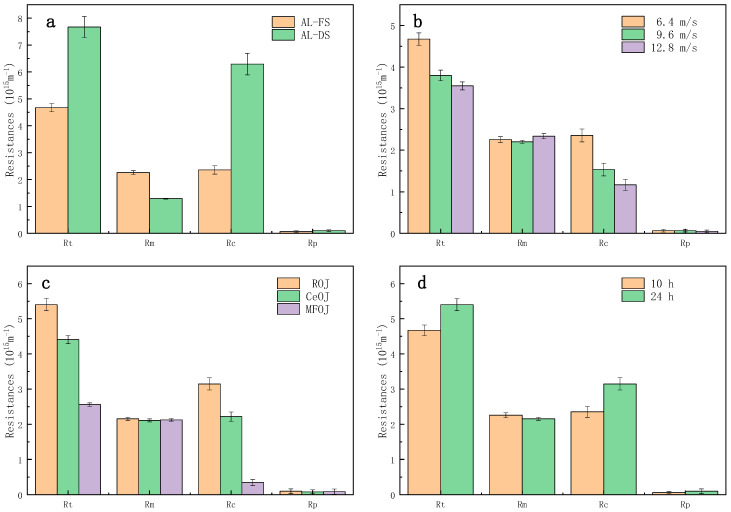
Comparison of process resistance of different (**a**) membrane orientations, (**b**) shear rates, (**c**) pretreatments and (**d**) running durations.

As shown in Figure 5a, from the perspective of process resistance in different membrane orientations, the total resistance R_t_ of the AL-DS mode was larger than that of the AL-FS mode, which explains why the water flux of the AL-DS mode was lower [28]. The membrane resistance R_m_ was greater on the AL-FS mode, but the fouling layer resistance *R_c_* on the AL-DS mode was much higher than that on the AL-FS mode. Both of them had a high recovery rate after cleaning, which indicated that the forward osmosis fouling was easy to clean and the irreversible fouling was neglectable. The resistance of the fouling layer accounted for 50.40% and 81.98% of the total resistance.

As shown in Figure 5b, increasing the cross-flow rate could effectively reduce the total resistance R_t_ by reducing the resistance of the fouling layer *R_c_*, since the high shear rate was able to prevent the pollutants from adhering to the membrane surface; thus, the fouling layer was difficult to grow.

As shown in Figure 5c, the pretreatment, both centrifugation and microfiltration, can effectively reduce the resistance of the fouling layer R_c_. Especially, the resistance of the fouling layer produced by the MFOJ was extremely low, consolidating the remark of Section 3.1 that the major foulants were eliminated by microfiltration.

Figure 5d shows a comparison of the process resistance between two different concentration-running durations. Although the duration was prolonged by 140%, the fouling layer resistance *R_c_* only increased by 33.62%, which indicated that the fouling growth was relatively slow after the formation of the first fouling layer, and the growth of membrane fouling mainly occurred in the initial stage of concentration.

### 3.3. Foulants/Membrane Interaction

In order to explore the effect of the membrane orientation and pretreatment on the interaction between the FS and the membrane surface, the xDLVO theory was used to analyze the experimental results.

The contact angles of the three probe liquids on the corresponding materials are shown in Table 1. The water contact angle of the active separation layer was comparable to that of the porous support layer. The water contact angle of the ROJ, CeOJ and MFOJ decreased in turn, implying that the pretreatment of centrifugation and microfiltration had a crucial effect on the hydrophilicity of feed solutes.

As shown in Table 2, the γ^LW^ of the two orientations of the membranes and the different feed solutes were obviously higher than γ^AB^, suggesting that the surface free energy of membranes and feed solutes was mostly contributed by the nonpolar Lifshitz–van der Waals forces. As for the acid and base components, γ^−^ of each sample was higher than γ^+^, representing a stronger Lewis base property of membranes. In general, the properties of electron donors were dominant on the surface of the organic polymer films due to the existence of various functional groups [36,37]. Combined with the contact angles between water and each sample, it can be claimed that the stronger the γ^−^ in the surface free energy, the better the wetting effect was, which is consistent with previous studies [38,39].

The value of ΔG_coh_ reflected the free energy per unit area between two contacting surfaces of the same material in water, which provided a quantitative presentation for the hydrophobicity/hydrophilicity of the membrane surface or feed solutes [40]. The material with positive values of ΔG_coh_ showed hydrophilic surface properties. Conversely, the material with negative values of ΔG_coh_ had hydrophobic surfaces [41]. According to the values of ΔG_coh_ listed in Table 3, the CTA membrane used in this study was hydrophobic, while the porous support layer showed stronger hydrophobicity. All the feed solutes were hydrophobic and the hydrophobicity was weakened after pretreatment, especially after microfiltration, resulting in a dramatical drop of ΔG_coh_ from −48.11 to −2.68 kT.

The summation of the Lifshitz–van der Waals force energy (ΔG^LW^), acid–base interaction energy (ΔG^AB^) and electrostatic force energy (ΔG^EL^) was defined as free energy of adhesion (ΔG_adh_). The ΔG_adh_ represents the interfacial free energy per unit area between feed solutes and membranes at contact in the water. The value of ΔG_adh_ can be used to evaluate the adsorption potential between feed solutes and membrane surface [42,43]. As shown in Table 4, the values of ΔG_adh_ between feed solutes and CTA membranes were negative for all conjugations, indicating attractions of different magnitudes between feed solutes and CTA membranes.

In different membrane orientations, the values of ΔG_adh_ between the active separation layer and feed solutes (FOAL-ROJ) were slightly lower than those between the porous support layer membrane and feed solutes (FOSL-ROJ). Therefore, the main difference of membrane fouling behavior between the AL-DS mode and the AL-FS mode may have resulted from the blockage of the porous support layer rather than the adsorption force between feed solutes and membranes.

The ROJ had a stronger hydrophobicity with a higher value of ΔG_coh_ compared with the CeOJ and MFOJ; thus, the ROJ was supposed to have a higher interaction energy with the CTA membranes at contact, which was confirmed by the higher value of ΔG_adh_. The membrane fouling produced by the MFOJ was the lightest, which can be explained by the minimum adhesion free energy as shown in Table 4.

**Figure 6:**
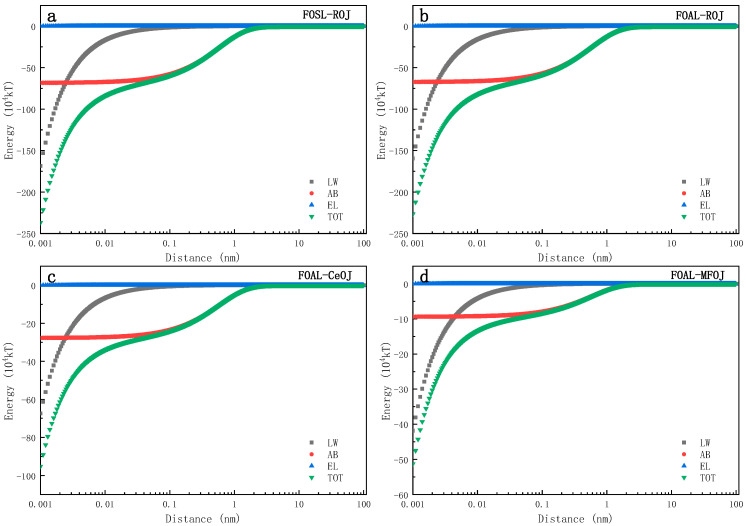
Evolutions of interaction energies between membrane and feed solutes over interfacial distance includes (**a**) FOSL–ROJ, (**b**) FOAL–ROJ, (**c**) FOAL–CeOJ and (**d**) FOAL–MFOJ.

Figure 6 shows the evolution of different interfacial interaction energies (LW, AB, EL, TOT) between the membrane surface and the feed solutes over a distance. It can be seen from the figure that there was no interaction between them when their distance was beyond 3 nm, while the interfacial interaction energy increased rapidly when the feed solute was approaching to the membrane surface within 3 nm. EL is a long-distance force, and its interfacial interaction energy had little impact on the total interfacial interaction energy, which is consistent with previous research [44,45]. The evolution curves also indicate that the LW and AB interface interaction could jointly affect the interface interaction between feed solutes and the membrane surface.

According to the xDLVO theory, if the interfacial interaction energy is positive, then the repulsive effect governs the interaction between the two interfaces which hinders the adsorption and accumulation of feed solutes on the membrane surface; otherwise, the negative interfacial energy indicates that the adsorption of feed solutes takes place on the membrane surface [30]. When the distance between the membrane surface and the feed solutes interface was 0.158 nm~3 nm, the polar interaction energy of AB was the dominant factor controlling the interface free energy and its magnitude increased rapidly with the decrease in the distance between the two interfaces. Therefore, in this distance range (0.158~3 nm), the evolution trend of total interface interaction energy TOT was consistent with that of the AB interaction energy as shown in Figure 6. When d < 0.158 nm, LW began to dominate the interaction between membranes and feed solutes. With the continuous decrease in the distance, the total interfacial interaction energy increased in the negative direction, suggesting a continuous increase of the interfacial repulsive force. This figure also indicates that the interfacial interaction energy was barely impacted by the membrane orientation since the evolution trends were almost the same between Figure 6a,b. In addition, compared with the ROJ and CeOJ, MFOJ was shown with a lower interfacial energy with the active layer of the membrane (Figure 6c,d). The interfacial interaction energy of the MFOJ was the lowest among them, which was well consistent with our previous results showing that the membrane fouling was greatly alleviated by the pretreatment of microfiltration.

**Figure 7:**
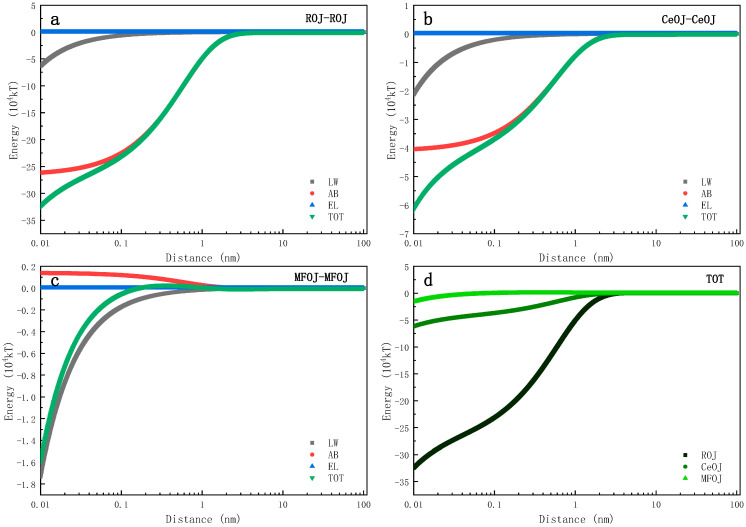
Evolutions of interaction energies between feed solutes over interfacial distance includes (**a**) ROJ–ROJ, (**b**) CeOJ–CeOJ, (**c**) MFOJ–MFOJ and (**d**) TOT.

Figure 7 shows the evolution of the interfacial interaction energy between one feed solute and another over a distance in the FO process for concentration. The evolution trends of the interaction energy of ROJ–ROJ and CeOJ–CeOJ were quite similar (Figure 7a,b) and always in the negative range, which indicates that the attractive force dominated those involved interfaces, implying that the later-coming solutes were easy to attach to the settled one, resulting in an easy growth scenario of membrane fouling. On the contrary, the evolution of the interaction energy for MFOJ–MFOJ (Figure 7c) showed distinctive features. When the distance of interface was 0.16 nm~1.50 nm, the total interfacial interaction energy was positive, indicating the repulsive force between feed solutes. In fact, the AB interaction energy was always positive within 1 nm in this case, leading to an important decrease in the total interaction energy (towards zero) compared to that of the ROJ or CeOJ, as shown in Figure 7d. Such revelation suggests that the pretreatment of microfiltration can effectively prevent the fouling layer from growing.

### 3.4. Foulant Identification

The ATR-FTIR was analyzed to determine the organic compositions of foulants on the membrane surfaces. The spectrums of pristine and fouled membranes were obtained.

**Figure 8:**
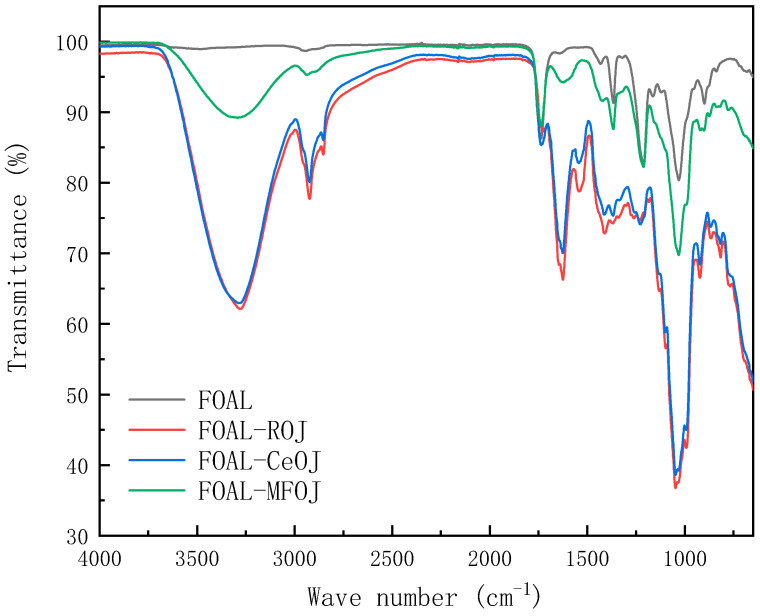
FTIR spectra of clean and fouled CTA membranes.

Figure 8 depicts the ATR-FTIR spectrum of the pristine membrane, on which five specific peaks can be obviously observed at the locations of 1736 cm^−1^, 1367 cm^−1^, 1212 cm^−1^, 1047 cm^−1^ and 900 cm^−1^. Signals at 736 cm^−1^, 1212 cm^−1^ and 1047 cm^−1^ corresponded to –C=O, –C–C–O– and –C–O groups, respectively, representing the characteristics of CTA FO membranes [35,46].

After the juice concentration experiments, certain specific peaks on the pristine CTA FO membrane decreased or disappeared. Other new absorption peaks emerged, especially after the fouling of the ROJ and CeOJ, indicating that the fouling layer had been settled on the surface of the FO membrane.

An amount of 3279 cm^−1^ represented the stretching and vibration of the O–H bond in the hydroxyl functional groups, and the sharp peak at 2923 cm^−1^ related to the stretching of the C–H bond was observed [47]. These adsorption peaks indicated the presence of polysaccharides or polysaccharide-like substances in the fouling layer. Furthermore, the peak at 1031 cm^−1^ was also attributed to polysaccharides or polysaccharide-like substances [48]. Moreover, two sharp absorbance peaks appeared at 1635 cm^−1^ and 1545 cm^−1^ corresponding to amides I (C=O stretching) and II (N–H in plane), indicating that the membrane surface contaminants contained amino and carboxyl functional groups, representing the existence of proteins or protein substances [49]. The spectrum of the MFOJ basically retained the original characteristic peak of the pristine membrane. Compared with the fouling of the ROJ and CeOJ, the absorption peak of the MFOJ decreased obviously at 3279 cm^−1^, and there was only a weak absorption peak at 2923 cm^−1^ and 1635 cm^−1^.

The results showed that the organic pollutants formed on the membrane surface of the ROJ and CeOJ were mainly polysaccharides and proteins, and microfiltration could greatly reduce the fouling of polysaccharides and remove almost all protein fouling.

## 4. Conclusions

In this work, the membrane fouling behaviors of the FO process for orange juice concentration were systematically investigated. The effects of different membrane orientations, shear rates and pretreatments on water fluxes and concentration factors were evaluated. The results showed that the AL-FS mode was more suitable for concentrating orange juice. The increase in the cross-flow rate made it difficult for pollutants to adhere to the membrane surface and the membrane fouling layer to grow; thus, reducing the resistance of the fouling layer, so as to improve the water flux and concentration efficiency. ATR-FTIR analysis revealed that the fouling layer of orange juice was mainly composed of proteins and polysaccharides. There was a strong attractive interaction between these pollutants and the FO membrane. Microfiltration pretreatment greatly reduced the content of the main pollutants, weakened the interaction of pollutants–membrane and pollutants–pollutants, and effectively prevented the growth of the fouling layer, which then lowered the resistance of the fouling layer. As a result, the water flux and the concentration efficiency of FO were greatly improved after such pretreatment.

## Figures and Tables

**Table 1:** The contact angle of the membranes and the feed solutes by three probe liquids.

Probe Liquids	Contact Angle (°)		
	Water	Formamide	Diiodomethane
FOSL	86.48 ± 3.77	57.54 ± 3.53	37.65 ± 2.25
FOAL	83.95 ± 3.33	50.55 ± 2.17	40.00 ± 2.03
ROJ	80.13 ± 2.48	62.18 ± 2.15	47.75 ± 2.12
CeOJ	69.12 ± 2.49	61.21 ± 2.17	53.25 ± 3.41
MFOJ	51.88 ± 2.92	39.12 ± 2.22	46.55 ± 3.28

**Table 2:** Surface tension (mJ/m^2^) of the membrane and the feed solutes.

	γ^LW^	γ^+^	γ^−^	γ^AB^	γ^TOT^
FOSL	40.77	0.18	1.74	1.13	41.90
FOAL	39.61	1.04	1.27	2.30	41.91
ROJ	35.52	0.03	7.56	1.02	36.54
CeOJ	32.45	0.01	19.27	1.07	33.51
MFOJ	36.17	0.98	26.09	10.11	46.28

**Table 3:** Cohesion free energy (mJ/m^2^) of membrane and feed solutes.

	ΔG^LW^	ΔG^AB^	ΔG^EL^	ΔG_coh_
FOSL	−5.89	−68.98	3.73 × 10^−13^	−74.87
FOAL	−5.28	−63.20	1.38 × 10^−12^	−68.48
ROJ	−3.33	−44.77	1.25 × 10^−12^	−48.11
CeOJ	−2.11	−13.01	1.13 × 10^−12^	−15.12
MFOJ	−3.62	0.94	5.80 × 10^−12^	−2.68

**Table 4:** Adhesion free energy (mJ/m^2^) between the membrane and the feed solutes.

	ΔG^LW^	ΔG^AB^	ΔG^EL^	ΔG_adh_
FOSL-ROJ	−4.43	−57.57	−2.48 × 10^−7^	−62.00
FOAL-ROJ	−4.20	−56.69	−4.37 × 10^−7^	−60.89
FOAL-CeOJ	−3.34	−43.97	−4.16 × 10^−7^	−47.30
FOAL-MFOJ	−4.37	−31.37	−3.11 × 10^−7^	−35.75

## Data Availability

All data presented in this study are available in the current article.
